# Polyglutamine expansion affects huntingtin conformation in multiple Huntington’s disease models

**DOI:** 10.1038/s41598-017-05336-7

**Published:** 2017-07-11

**Authors:** Manuel Daldin, Valentina Fodale, Cristina Cariulo, Lucia Azzollini, Margherita Verani, Paola Martufi, Maria Carolina Spiezia, Sean M. Deguire, Marta Cherubini, Douglas Macdonald, Andreas Weiss, Alberto Bresciani, Jean-Paul Gerard Vonsattel, Lara Petricca, J. Lawrence Marsh, Silvia Gines, Iolanda Santimone, Massimo Marano, Hilal A. Lashuel, Ferdinando Squitieri, Andrea Caricasole

**Affiliations:** 1 IRBM Science Park, Via Pontina km 30.600, 00071 Pomezia, Rome Italy; 2 IRBM Promidis, Via Pontina km 30.600, 00071 Pomezia, Rome Italy; 3 Laboratory of Molecular and Chemical Biology of Neurodegeneration, Brain Mind Institute, Station 19, School of Life Sciences, Ecole Polytechnique Fédérale de Lausanne (EPFL), CH-1015 Lausanne, Switzerland; 4 Departamento de Ciencias Biomedicas, Facultat de Medicina, Instituto de Neurociencias, Universitat de Barcelona, Barcelona, Spain; 5 CHDI Management/CHDI Foundation, Los Angeles, CA 90045 USA; 6 Taub Institute for Research on Alzheimer’s disease and the Aging Brain, Columbia University Medical Center, 710 West 168th Street, New York, NY 10032 USA; 7 Department of Developmental and Cell Biology, University of California, Irvine, 92697 USA; 8 Huntington and Rare Diseases Unit, IRCCS Casa Sollievo della Sofferenza, San Giovanni Rotondo, Italy; 9 Evotec AG, Manfred Eigen Campus, Hamburg, Germany

## Abstract

Conformational changes in disease-associated or mutant proteins represent a key pathological aspect of Huntington’s disease (HD) and other protein misfolding diseases. Using immunoassays and biophysical approaches, we and others have recently reported that polyglutamine expansion in purified or recombinantly expressed huntingtin (HTT) proteins affects their conformational properties in a manner dependent on both polyglutamine repeat length and temperature but independent of HTT protein fragment length. These findings are consistent with the HD mutation affecting structural aspects of the amino-terminal region of the protein, and support the concept that modulating mutant HTT conformation might provide novel therapeutic and diagnostic opportunities. We now report that the same conformational TR-FRET based immunoassay detects polyglutamine- and temperature-dependent changes on the endogenously expressed HTT protein in peripheral tissues and post-mortem HD brain tissue, as well as in tissues from HD animal models. We also find that these temperature- and polyglutamine-dependent conformational changes are sensitive to bona-fide phosphorylation on S13 and S16 within the N17 domain of HTT. These findings provide key clinical and preclinical relevance to the conformational immunoassay, and provide supportive evidence for its application in the development of therapeutics aimed at correcting the conformation of polyglutamine-expanded proteins as well as the pharmacodynamics readouts to monitor their efficacy in preclinical models and in HD patients.

## Introduction

Huntington’s disease (HD, MIM 143100) is a progressive, monogenic, autosomal dominant neurodegenerative disease caused by a CAG triplet repeat expansion, encoding polyglutamine (polyQ), within exon 1 of the *HTT* gene (MIM 613004)^1, 2^. This mutation leads to the generation of mutant HTT (mHTT) fragments, some of which contain the polyglutamine expansion, produced through mechanisms such as aberrant splicing or proteolysis^3, 4^. Amino-terminal mHTT fragments present a strong propensity to misfold and self-associate, giving rise to oligomers and the characteristic aggregates in relevant brain areas, and are sufficient to cause a pathology strongly reminiscent of HD in various animal models^5–9^. Misfolding of HTT amino-terminal fragments caused by the polyQ expansion is believed to play a key role in mHTT’s aggregation and toxicity, and indeed the expansion of the polyQ domain associated with the HD mutation was recently shown to alter the conformation of the carboxyl-term region of HTT^10–12^. Interestingly, phosphomimetic mutations at specific residues can at least partially ameliorate the conformational rigidity imposed on HTT by the mutant polyQ expansion^10^, and critically affect the subcellular localization, stability, aggregation and toxicity properties of mHTT^13–18^. The modification of residues associated with phosphorylation has been demonstrated to critically affect the toxic properties of huntingtin (S13/S16D; Gu *et al*.^15^) and other polyQ disease-associated proteins (S776 in Ataxin 1^19, 20^; S215 and S792)^21^ in mice, thus suggesting that phosphorylation can profoundly affect pathology *in vivo*. Collectively, these datasets suggest that increasing HTT N17 phosphorylation might be a viable therapeutic approach aimed at ameliorating the toxic properties of mHTT through conformational modulation. However, addressing this hypothesis requires suitable assays to identify genetic and/or pharmacological modulators of HTT N17 phosphorylation, as well as to monitor HTT conformation in different HD models and, ultimately, in HD patients.

Various immunoassays, ranging from sandwich-type immunoassays^22–24^ to time-resolved Forster resonance energy transfer (TR-FRET) based assays^25, 26^ can inform on HTT levels in different sample types. However, studying HTT conformation remains a challenge and is predominantly addressed using biophysical approaches. Using purified HTT protein fragments and lysates from cells expressing exon 1 (residues 1–90), N548 (residues 1–548) or full length (FL, residues 1–3144) HTT proteins bearing wild type or mutant polyQ expansions, we recently reported a temperature-dependent, polyQ-dependent, reversible conformational change imparted on HTT by the polyQ expansion, as detected by TR-FRET immunoassays and confirmed by circular dichroism^11^. Similar findings were reported by others^12^. Interestingly, we also noticed the temperature- and polyQ-dependent conformational change using analogous TR-FRET assays interrogating the polyQ domain of other CAG repeat proteins, underscoring the general relevance of the methodology for CAG repeat protein misfolding diseases^27^.

TR-FRET immunoassay detection is based on the labeling of an antibody pair with a rare earth ion fluorophore donor and an acceptor fluorophore, thereby producing a specific TR-FRET signal when the donor and acceptor labeled antibodies bind to their antigen simultaneously^28^. The used antibody pair comprised previously characterized monoclonals (2B7 and MW1) interrogating two regions of known relevance for HTT conformation and biological properties: the N17 domain and the polyQ domain^29–33^. Structurally, the conformational immunoassay readout was associated with temperature- and polyQ-dependent variations in the α-helicity content in the Amino-terminal region of HTT^11, 12^. Indeed, cooling increased α-helicity more strongly for mutant than for wild type HTT, an effect which is exquisitely polyQ dependent^11^. Potentially, the rapid, scalable and homogeneous conformational TR-FRET-based immunoassay provides a useful screening tool to identify genetic and pharmacological modulators of mHTT conformation. However, interrogation of the utility of the conformational TR-FRET-based immunoassay for therapeutic development requires in the first instance demonstration of its translational relevance to mHTT protein in animal models of HD and in clinical samples.

We therefore investigated the capacity of the conformational immunoassay to detect mHTT conformation in selected preclinical models and in clinical samples (fibroblasts and lymphocytes from HD patients and post-mortem HD brain tissue), and found that the assay can efficiently detect the conformational constraint imposed by polyQ expansion in these samples. We also examined the capacity of bona-fide HTT N17 phosphorylation, rather than phosphomimetic mutations, to modulate HTT conformation as measured by the conformational immunoassay in purified, synthetic HTT exon 1 proteins bearing phosphorylated S13 and S16 residues. We find that compound S13/S16 phosphorylation (but not the corresponding individual modifications) can at least partially revert the conformational rigidity imposed by polyglutamine expansion on HTT.

## Results

### The conformational immunoassay preferentially reports on mutant huntingtin conformational behavior in mixed samples containing both wild type and mutant Huntingtin proteins

**Figure 1:**
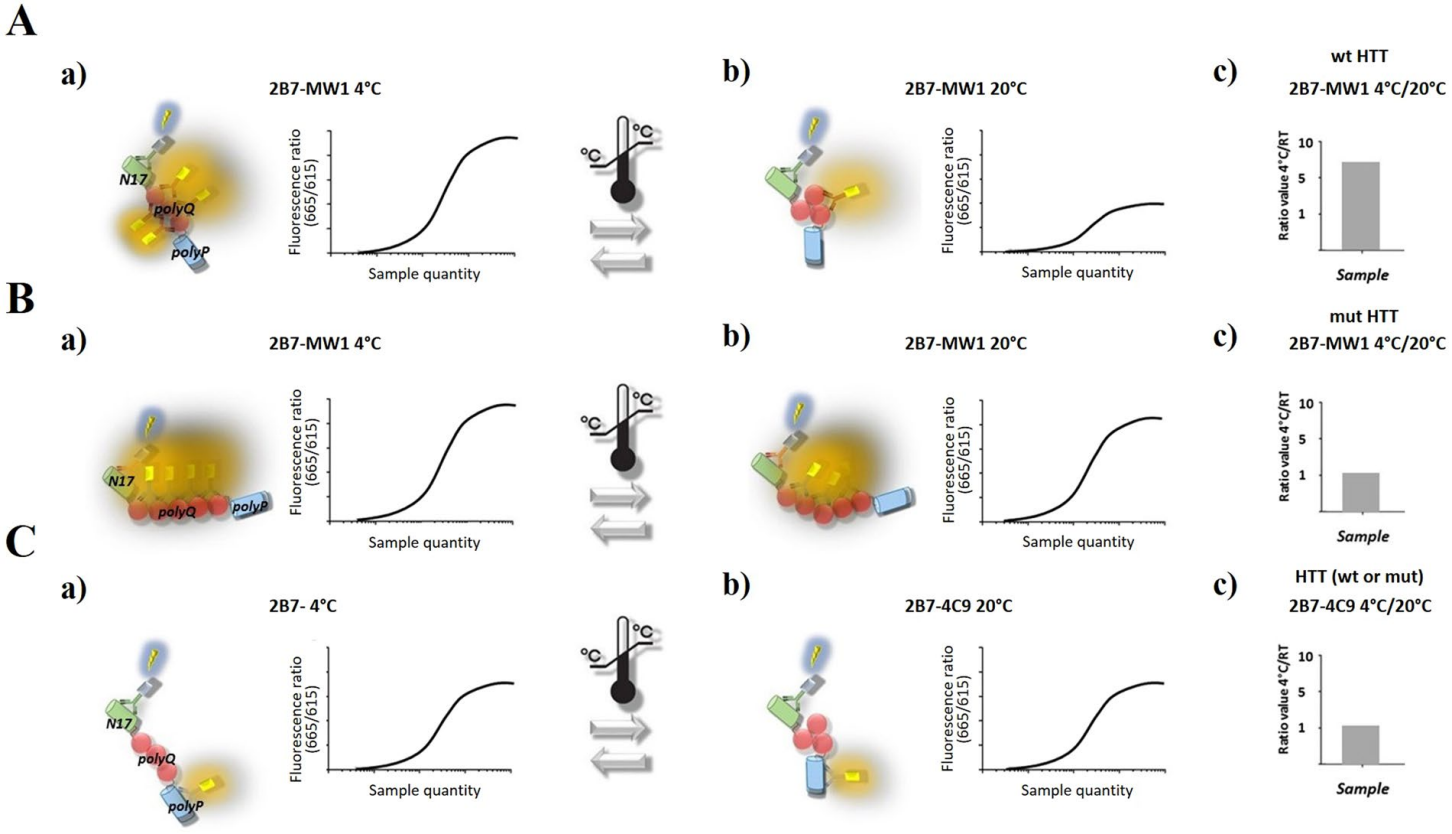
A schematic model illustrating the 2B7/MW1 conformational TR-FRET immunoassay for detection of HTT amino-terminal conformation (elaborated from ref. 11). The principal domains of HTT exon 1 are indicated (N17, polyQ and polyproline), together with the identity of the corresponding monoclonal antibodies used for TR-FRET detection (2B7, MW1 and 4C9, respectively). (**A**) The TR-FRET antibody pair (2B7/MW1) including one antibody targeting the polyQ domain (MW1) engages polyQ epitopes on wild type HTT differently depending on incubation temperature, as the wild type polyQ repeat is flexible and can adopt different conformations (a and b). This results in a higher ratio (≫1) between TR-FRET signals obtained from the same sample at the different temperatures (c). (**B**) The mutant polyQ repeat in mHTT is less flexible and availability/position of the polyQ epitopes for detection by the same TR-FRET antibody pair is relatively invariant at different temperatures (a and b), resulting in ratio between TR-FRET signals obtained from the same sample at the different temperatures close to 1(c). (**C**) Instead, a TR-FRET antibody pair (2B7/4C9) which does not include one antibody targeting the polyQ domain does not interrogate changes in polyQ conformation, does not significantly discern between wild type and mutant HTT and produces a ratio between TR-FRET signals obtained from the same sample at the different temperatures close to 1 (c).

Previously, we and others reported novel immunoassays capable of detecting temperature- and polyQ-dependent, conformational changes in bacterially produced, purified HTT proteins and in lysates of cells overexpressing either wild type or mHTT^11, 12^. The conformational immunoassay is based on a TR-FRET readout employing antibodies interrogating two distinct epitopes of HTT, including one within the polyQ region (e.g. 2B7, specific for the N17 region of HTT, and MW1, recognizing a polyQ-dependent epitope; refs 25 and 26) See Fig. 1A for a schematic model representation. A change in the incubation temperature leads to a change in TR-FRET signal, an effect likely due to conformational flexibility^11, 12^. Expansion of the polyQ repeat impairs this conformational flexibility, reducing the temperature-dependent modulation of TR-FRET signal in a polyQ-dependent fashion (Fig. 1B; refs 11 and 12). Using an antibody combination where the polyQ domain is not interrogated by one of the antibodies in the TR-FRET IgG pair (for instance 2B7 and 4C9, this latter specific for an epitope within the polyproline region; Fig. 1C) results in an immunoassay where the TR-FRET signal is not sensitive to temperature changes and polyQ expansion.

**Figure 2:**
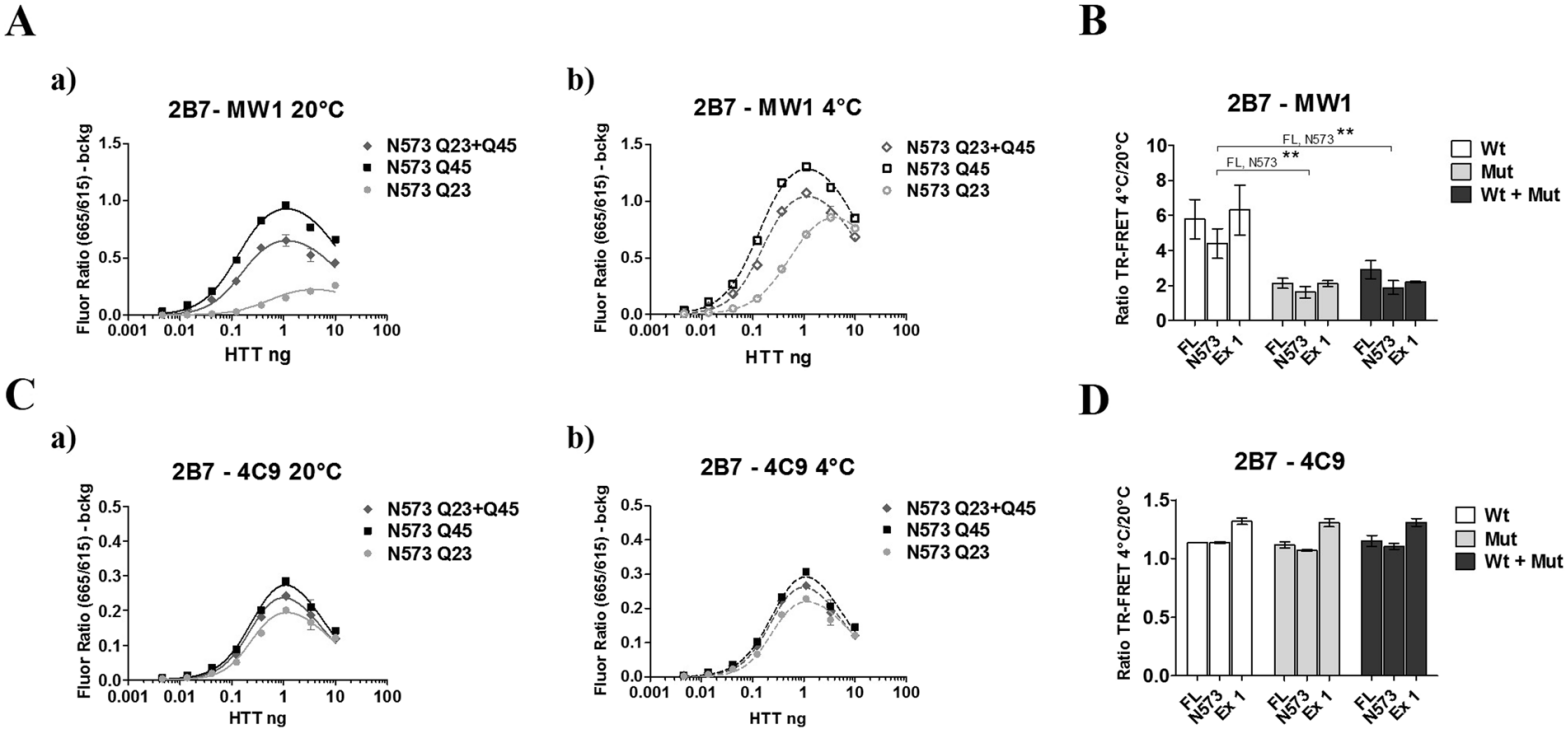
The 2B7/MW1 conformational immunoassay preferentially detects mHTT conformation in cocktails of isolated HTT proteins with wild type or mutant polyQ. (**A**) The 2B7/MW1 TR-FRET assay performed on wild type HTT, mHTT or a cocktail (of equivalent final protein concentration) of the two proteins at 20 °C (a) or measured after shifting the same samples to 4 °C (b). For simplicity, only results for a representative experiment performed on the N573 HTT protein fragments are shown. (**B**) Summary of data (ratio TR-FRET signals at 4 °C/20 °C) obtained on individual isolated HTT proteins (3 lengths, namely exon 1, N573 and full length) or cocktails of equivalent final protein concentration (Wt + Mut). (**C**) Same as A, obtained on the same proteins using a (control) TR-FRET immunoassay (2B7/4C9) which does not interrogate the polyQ region and does not detect a temperature- and polyQ dependent conformational change^11, 12^, demonstrating efficient detection of all proteins. (**D**) Same as B (ratio TR-FRET signals at 4 °C/20 °C) obtained on the same proteins using a (control) TR-FRET immunoassay (2B7/4C9) which does not interrogate the polyQ region and does not detect a temperature- and polyQ dependent conformational change. In (B and D) values represent means and standard deviations of the means of three independent experiments (two-way ANOVA with Bonferroni’s post-test, degrees of significance are indicated).

However, the experiments previously reported did not interrogate the behaviour of the conformational immunoassay under conditions compatible with allelic heterozygosity, where both wild type and mHTT are expressed (such as in heterozygous mutant HD animal models and in clinical HD). Effectiveness in this setting is essential if the assays are to be useful in the development of therapeutic approaches aimed at modulating mHTT conformation. We therefore proceeded to monitor temperature- and polyQ-dependent conformation of HTT proteins when both wild type and mHTT are present, in samples of increasing complexity and translational relevance. We started with the simplest context, represented by purified, isolated proteins. The 2B7/MW1 and 2B7/4C9 TR-FRET assays^11^ were carried out to interrogate equimolar cocktails of wild type (normal polyQ length) and mutant (expanded polyQ length) HTT proteins, in comparison with samples containing either the individual wild type or the mutant proteins. Purified semisynthetic Exon 1 proteins with a Q23 or Q43 polyQ repeat^34^ or purified recombinant N573 fragments, Q23 and Q45^23^, and full length HTT Q16 and Q46 were used^35^. As the conformational immunoassay is dependent on interrogating the polyQ region with specific antibodies^11^, we expected that the signal resulting from engagement of the mutant protein would be relatively higher than that resulting from engagement of the wild type HTT protein owing to the presence of a larger number of epitopes for the polyQ-specific antibody^30, 32, 36–38^. As expected, the 2B7/MW1 TR-FRET signal obtained from the mHTT protein is significantly higher than that obtained on the wild type HTT protein, and in the wild-type and mutant protein cocktail this prevails (Fig. 2Aa; shown here for the N573 recombinant proteins only, for simplicity). When the samples are shifted to 4 °C, the expected polyQ-dependent change in signal was observed, with the wild type HTT protein fragment yielding a larger relative change than the mutant HTT protein fragment (Fig. 2Ab). Significantly, in the wild-type and mHTT protein cocktails the resulting TR-FRET values are comparable to those obtained with the individual mHTT proteins, presumably owing to the higher 2B7/MW1 signal obtained with the mutant protein (Fig. 2Aa,b and B), and this is true for all three purified HTT proteins examined (full length, N573 and exon 1; Fig. 2B). The 2B7/4C9 TR-FRET immunoassay did not reveal any temperature- and polyQ-dependent effects (Fig. 2Ca and b), and this is observed for all three HTT protein lengths (Fig. 2D). Therefore, the conformational immunoassay preferentially detects the behavior of the mHTT protein when both wild type and mutant proteins are present in the sample in a purified form, likely owing to the higher apparent affinity of the anti-polyQ antibody (MW1) for expanded polyglutamine repeats.

**Figure 3:**
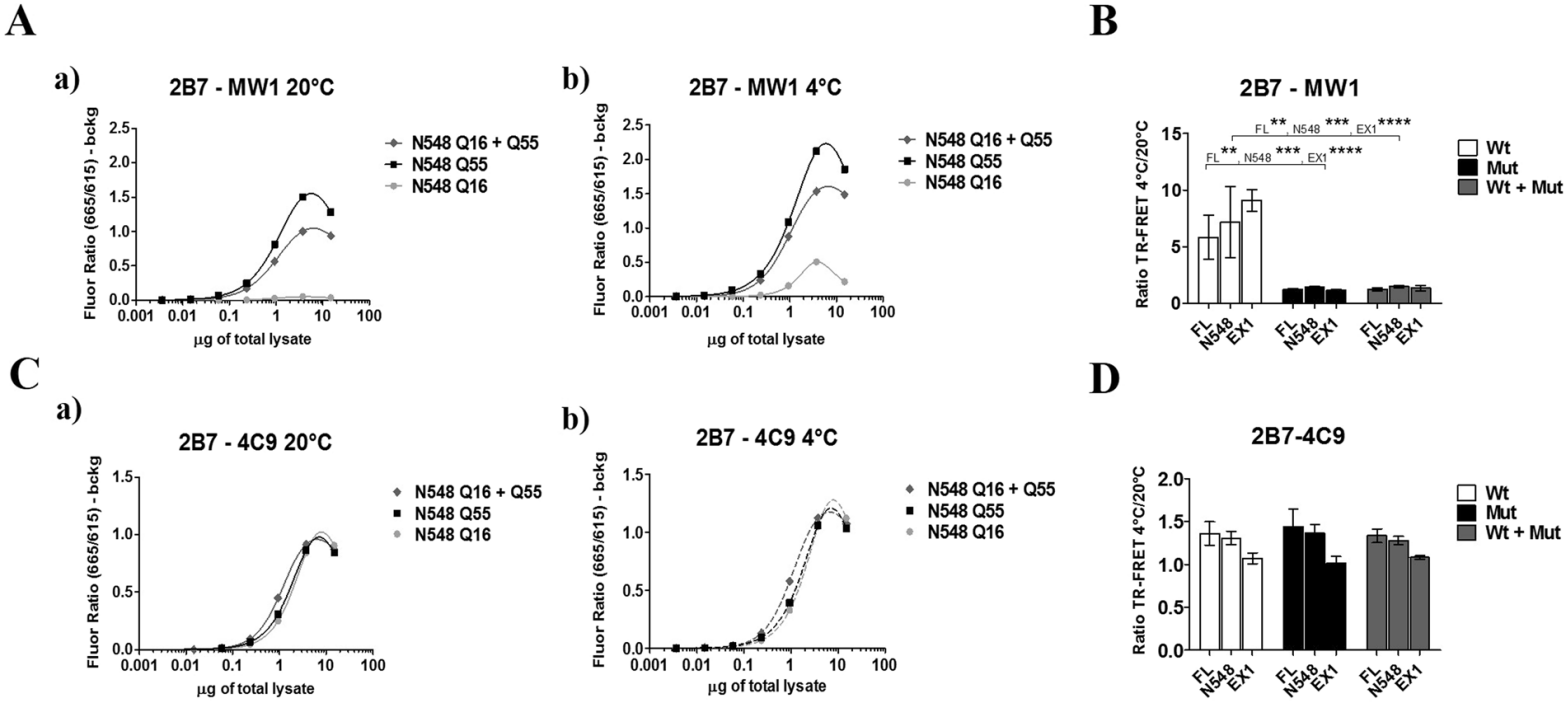
The 2B7/MW1 conformational immunoassay preferentially detects mHTT conformation in lysates of HEK293T cells transfected with plasmids encoding wild type and mutant HTT proteins. (**A**) The 2B7/MW1 TR-FRET assay performed on cell lysates expressing wild type HTT, mutant HTT or a cocktail of the two proteins (produced by co-transfecting equivalent concentrations of plasmid constructs encoding wild type and mutant HTT proteins) at 20 °C (a) or measured after shifting the same samples to 4 °C (b). For simplicity, only results for a representative experiment performed on lysates transfected with constructs encoding N548 HTT proteins are shown. (**B**) Summary of data (ratio TR-FRET signals at 4 °C/20 °C) obtained on lysates of HEK293T cells transfected with plasmids encoding wild type and mutant HTT proteins (3 lengths, namely exon 1, N548 and full length) or cocktails of plasmids encoding wild type and mutant HTT of equivalent concentration (Wt + Mut). (**C**) Same as A, obtained on the same lysates using a (control) TR-FRET immunoassay (2B7/4C9) which does not interrogate the polyQ region and does not detect a temperature- and polyQ dependent conformational change^11, 12^, demonstrating efficient detection of all proteins. (**D**) Same as B (ratio TR-FRET signals at 4 °C/20 °C) obtained on the same lysates using a (control) TR-FRET immunoassay (2B7/4C9) which does not interrogate the polyQ region and does not detect a temperature- and polyQ dependent conformational change. In (B and D) values represent means and standard deviations of the means of three independent experiments (two-way ANOVA with Bonferroni’s post-test, degrees of significance are indicated).

**Figure 4:**
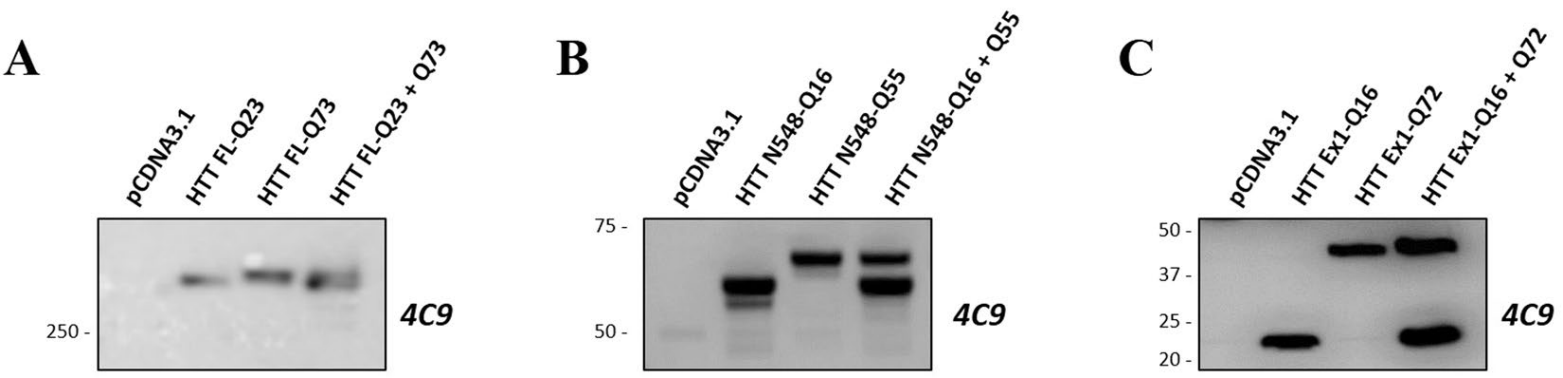
Representative Western blotting experiments of HEK293T cell lysates obtained by transient transfection with plasmids encoding HTT proteins, probed with antibody 4C9 (detecting HTT). (**A**) Western blot of HEK293T cell lysates transfected with either individual full length HTT proteins, wild type or mutant, or a cocktail of plasmids encoding both (of equivalent DNA concentration); samples were loaded on 6% Gel in order to better resolve the two full lengths proteins. (**B**) Western blot of HEK293T cell lysates transfected with either individual N548 HTT proteins, wild type or mutant, or a cocktail of plasmids encoding both (of equivalent DNA concentration). (**C**) Western blot of HEK293T cell lysates transfected with either individual exon 1 HTT proteins, wild type or mutant, or a cocktail of plasmids encoding both (of equivalent DNA concentration). In B and C samples were loaded on 4–12% gradient gel. The WB membranes in B and C were probed with anti-GAPDH antibody as loading control. GAPDH images are reported in supplementary information: Figs S2 and 3. It was not possible to test GAPDH as control in A because, the gel was run in order to resolve high molecular weight proteins, for distinguishing the wild type form from the mutant form of HTT. Indeed, the molecular weights included into the gel did not comprise proteins lower than 100 kDa (as GAPDH). All images were cropped from the original acquired file. Full-length blots are reported in Supplementary Information: Figures S1, S2 and S3.

Similar results to those observed using purified proteins were obtained using lysates of HEK293T cells transfected with expression plasmids encoding HTT Exon 1 (Q16 or Q72), HTT N548 (Q16 and Q55) or full length HTT proteins (Q23 and Q73), or co-expressing identical amounts of plasmid DNA encoding the wild type and the mutant HTT proteins. As observed when purified proteins were examined, HEK293T lysates expressing HTT proteins with an expanded (mutant) polyQ repeat yielded relatively higher 2B7/MW1 TR-FRET signals than cell lysates expressing wild type HTT (Fig. 3Ai and ii). In the wild-type and mutant HTT protein cocktails the resulting 2B7/MW1 TR-FRET values are comparable to those obtained from lysates expressing the individual mutant huntingtin proteins, again likely owing to the higher 2B7/MW1 signal obtained with the mutant proteins (Fig. 3Aa and b), and again this was observed irrespective of the length of the expressed HTT protein (Fig. 3B). The control analysis using the 2B7/4C9 antibody pair instead did not demonstrate any temperature- and polyQ-dependent effect (Fig. 3Ca,b and D). Correct expression of the different constructs was evaluated by Western blotting (Fig. 4). Therefore, under conditions modelling allelic heterozygosity in biological samples overexpressing wild type and mutant HTT proteins, the conformational immunoassay preferentially detects the behavior of the mutant (expanded polyQ) HTT.

**Figure 5:**
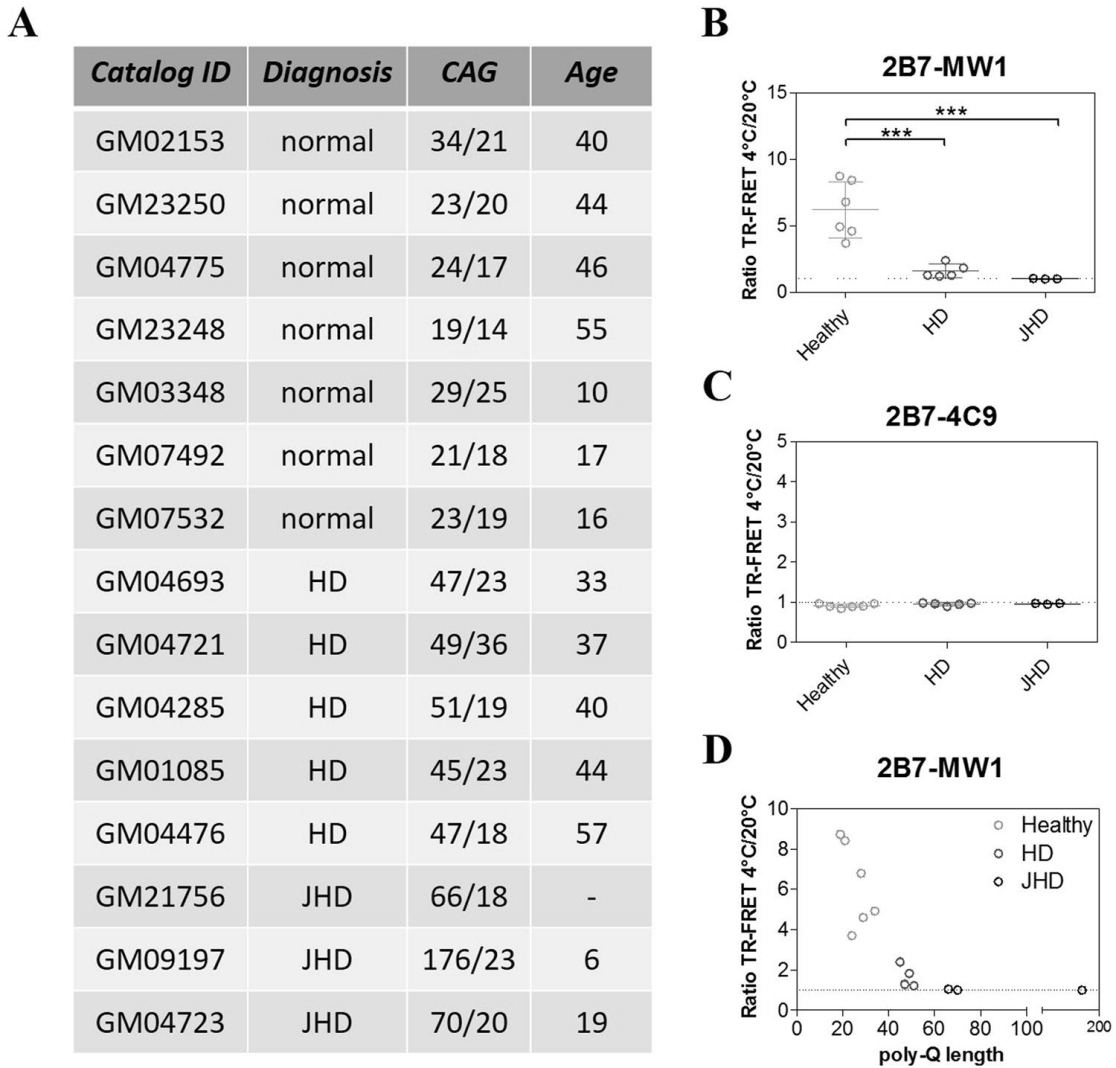
The 2B7/MW1 conformational immunoassay detects mHTT conformation in lysates of immortalized control and HD human fibroblasts (obtained from heterozygous HD donors). (**A)** Table illustrating the salient features of human control and HD immortalized fibroblasts used in this study. (**B**) Summary of 2B7/MW1 conformational immunoassay data (ratio TR-FRET signals at 4 °C/20 °C) obtained on lysates of control and HD fibroblasts. (**C**) Summary of 2B7/4C9 control immunoassay data (ratio TR-FRET signals at 4 °C/20 °C) obtained on the same lysates of control and HD fibroblasts. (**D**) Same as A, plotted against CAG (polyQ) repeat length rather than against control/HD genotype. In B and C values represent means and standard deviations of the means of three independent experiments (two-way ANOVA with Bonferroni’s post-test, degrees of significance are indicated).

Previously published data have not investigated HTT conformation when the protein is expressed at endogenous levels, reporting only on either individual purified proteins or lysates from cells overexpressing either wild type or mutant HTT^11, 12^. We proceeded to investigate the conformation of endogenously expressed HTT in cellular HD models where wild type and mutant HTT are expressed at normal levels from the endogenous locus. We analyzed cell lysates from HD patient-derived cells, namely immortalized primary fibroblasts from control and HD individuals, of known polyQ length status^39^. This immortalized fibroblast cell lysate sample set included 5 samples from control cells (polyQ < 35), 5 samples from HD cells (polyQ < 60), and 3 samples from cells bearing large expansions (polyQ > 60) representative of juvenile HD (Fig. 5A). We first investigated HTT levels in these samples using the 2B7/MW1 and 2B7/4C9 TR-FRET immunoassays and, as expected, due to increased apparent affinity of MW1 for expanded polyQ^25, 26^, the 2B7/MW1 assay revealed a polyQ-dependent signal (higher signal in HD fibroblasts relatively to control fibroblasts) while the 2B7/4C9 assay detected comparable HTT levels irrespective of polyQ length (data not shown). Consistent with data from overexpression cellular models presented above, the 2B7/MW1 TR-FRET conformational immunoassay detected a strong conformational difference between lysates from HD fibroblasts and control fibroblasts (Fig. 5B), with a clear polyQ-dependence (Fig. 5D). No such differences were observed with the control 2B7/4C9 TR-FRET immunoassay (Fig. 5C), consistent with the requirement to interrogate the polyQ region with one of the two antibody pairs^11, 12^. Collectively, these data indicate that the 2B7/MW1 TR-FRET based conformational immunoassay can effectively detect the conformational constraint imposed by polyQ expansion on HTT in human cellular models of HD, either under conditions where HTT is overexpressed or where it is expressed at endogenous levels from the relevant genomic locus.

### Conformational interrogation of mutant huntingtin conformation in animal models of HD

Having determined that the 2B7/MW1 TR-FRET based conformational immunoassay can effectively detect HTT conformation in cellular models of HD, we turned to an analysis of tissues from animal models of HD.

**Figure 6:**
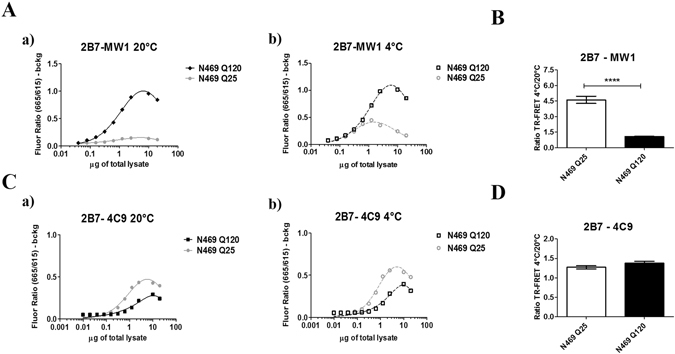
The 2B7/MW1 conformational immunoassay detects mHTT conformation in a Drosophila transgenic HD model. (**A**) The 2B7/MW1 TR-FRET assay performed on tissue homogenates of flies expressing a human wild type HTT N469 fragment or a mHTT N469 fragment at 20 °C (a) or measured after shifting the same samples to 4 °C (b). (**B**) Summary of data (ratio TR-FRET signals at 4 °C/20 °C) from three independent experiments. (**C**) Same as A, obtained on the same samples using a (control) TR-FRET immunoassay (2B7/4C9) which does not interrogate the polyQ region and does not detect a temperature- and polyQ dependent conformational change^11, 12^, demonstrating efficient detection of all proteins. (**D**) Same as B (ratio TR-FRET signals at 4 °C/20 °C) obtained on the same samples using a (control) TR-FRET immunoassay (2B7/4C9) which does not interrogate the polyQ region and does not detect a temperature- and polyQ dependent conformational change. In B and D values represent means and standard deviations of the means of three independent experiments (Datasets were tested for normality and Student’s t-test, degrees of significance are indicated).

The 2B7/MW1 TR-FRET conformational immunoassay, applied to brain tissue samples collected from the heterozygous mouse knock-in (KI) HD model (Q7/Q111)^40^, successfully detected the presence of mHTT and revealed the expected lack of conformational flexibility (data not shown). However, the TR-FRET assay did not detect HTT in knock-in mice harboring a humanized HTT exon 1 with a CAG_7_ repeat (Q7/Q7)^41^, as expected and previously reported^23^. Although we had no access to alternative mouse models expressing a wild type HTT with a polyQ length larger than Q7 and a corresponding mutant HTT protein, samples were available from a characterized Drosophila HD model expressing a large Amino-terminal (N469) fragment of human HTT with either Q25 or Q120^9^. HTT levels were investigated in these samples using the 2B7/MW1 and 2B7/4C9 TR-FRET immunoassays, and as expected the 2B7/MW1 assay revealed a polyQ-dependent signal (higher signal in animals expressing HTT with a mutant expansion) relative to flies expressing wild type HTT (Fig. 6Aa), while the 2B7/4C9 assay detected comparable HTT levels irrespective of polyQ length (Fig. 6Ca). Significantly, the 2B7/MW1 TR-FRET conformational immunoassay detected a strong conformational difference between lysates from HD Tg flies and control Tg flies (Fig. 6Aa,b and B). No such differences were observed with the control 2B7/4C9 TR-FRET immunoassay (Fig. 6Ca,b and D). These data indicate that the 2B7/MW1 TR-FRET based conformational immunoassay can effectively detect the conformational constraint imposed by polyQ expansion on HTT in animal models of HD.

### Interrogation of mutant huntingtin conformation in peripheral cells and post-mortem brain tissue from control and HD individuals

**Figure 7:**
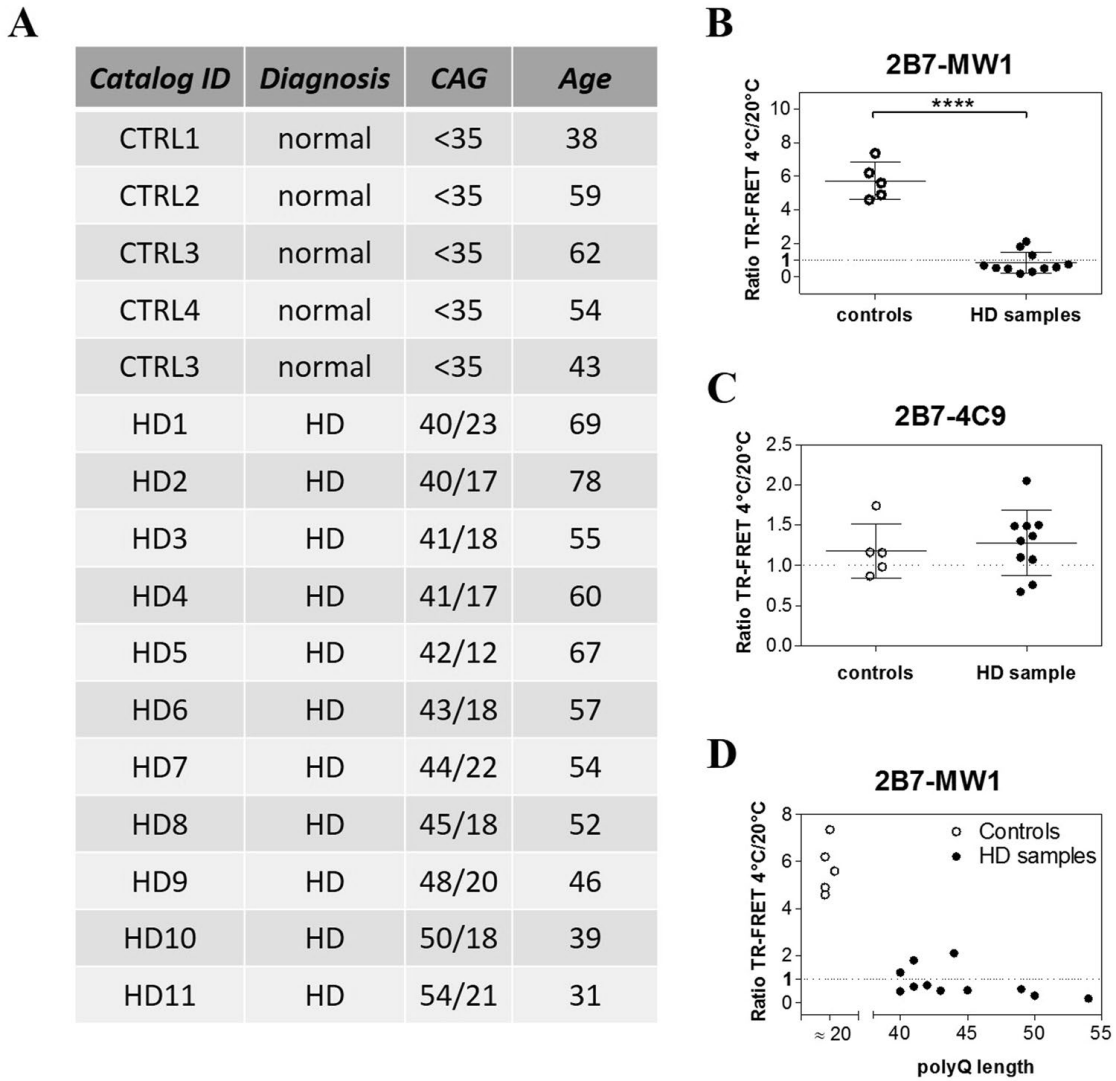
The 2B7/MW1 conformational immunoassay detects mHTT conformation in lysates of control and HD human PBMCs. (**A**) Table illustrating the salient features of human control and HD donors of samples for this study. (**B**) Summary of 2B7/MW1 conformational immunoassay data (ratio TR-FRET signals at 4 °C/20 °C) obtained on lysates of control and HD PBMCs. (**C**) Summary of 2B7/4C9 control immunoassay data (ratio TR-FRET signals at 4 °C/20 °C) obtained on the same lysates of control and HD fibroblasts. (**D**) Same as A, plotted against CAG (polyQ) repeat length rather than against control/HD genotype. In B and C values represent means and standard deviations of the means of three independent experiments (Datasets were tested for normality and Student’s t-test, degrees of significance are indicated).

**Figure 8:**
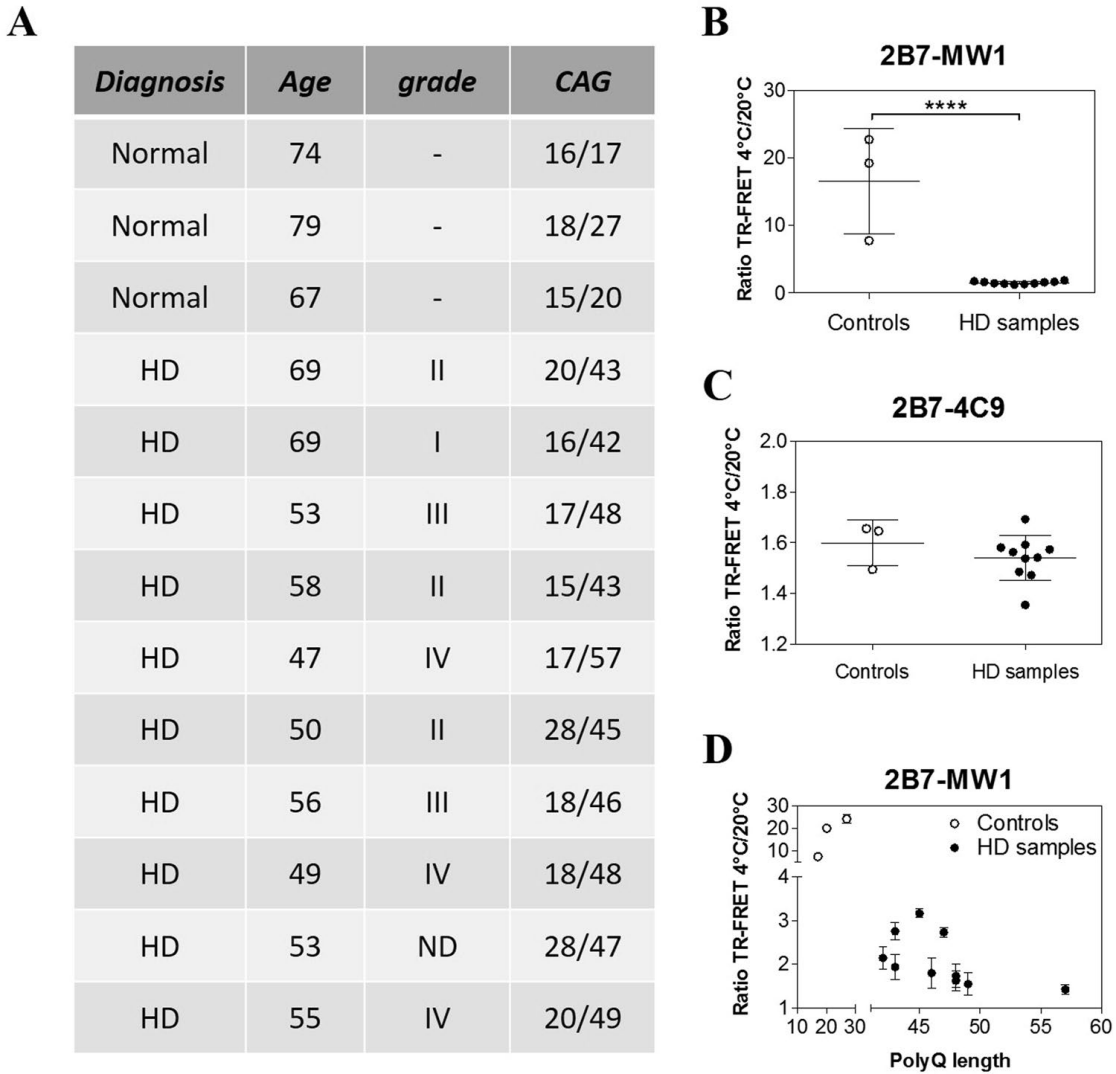
The 2B7/MW1 conformational immunoassay detects mHTT conformation in homogenates of control and HD post-mortem human brains (cortex). (**A**) Table illustrating the salient features of human control and HD donors of samples for this study. (**B**) Summary of 2B7/MW1 conformational immunoassay data (ratio TR-FRET signals at 4 °C/20 °C) obtained on homogenates of control and HD brain samples. (**C**) Summary of 2B7/4C9 control immunoassay data (ratio TR-FRET signals at 4 °C/20 °C) obtained on the same homogenates of control and HD brain samples. (**D**) Same as A, plotted against CAG (polyQ) repeat length rather than against control/HD genotype. In B and C values represent means and standard deviations of the means of three independent experiments (Datasets were tested for normality and Student’s t-test, degrees of significance are indicated). Signals obtained from TR-FRET immunoassays, preparatory for the conformational analysis, are reported in Supplementary Information: Figure S6.

Next, we sought to determine the potential translational value of the 2B7/MW1 TR-FRET conformational immunoassay, by interrogating biological samples of clinical relevance, namely samples of peripheral (lymphocytes) and central (post-mortem brain tissue) origin from control individuals or clinically and genetically characterized HD patients. Cell lysates of blood lymphocytes from healthy individuals (n = 5) and HD patients (N = 11; Fig. 7A) were analyzed for HTT protein levels with the 2B7/MW1 and 2B7/4C9 TR-FRET assays. Significantly, the 2B7/MW1 TR-FRET conformational immunoassay detected a strong conformational difference between lysates from HD lymphocytes and from control lymphocytes (Fig. 7B), with a clear polyQ-dependent effect (Fig. 7D). The control 2B7/4C9 TR-FRET immunoassay instead did not reveal changes in signal intensity dependent on polyQ length and temperature, as expected (Fig. 7C). Using the same TR-FRET immunoassays, we also investigated protein lysates from post-mortem brain tissue (frontal cortex) from control (n = 3) and clinically characterized HD individuals (n = 10; Fig. 8A). As expected, the 2B7/MW1 TR-FRET immunoassay revealed a higher signal in lysates from HD brain while the 2B7/4C9 TR-FRET immunoassay detected comparable levels of HTT protein in lysates from control and HD brain tissue (data showed in Supplementary Information: Figure S6). Notably, the 2B7/MW1 TR-FRET conformational immunoassay detected a strong conformational difference between lysates from HD brain and control brain (Fig. 8B), with a clear polyQ-dependent effect (Fig. 8D). The control 2B7/4C9 TR-FRET immunoassay instead did not reveal changes in signal intensity dependent on polyQ length and temperature (Fig. 8C). Given these results, we concluded that the 2B7/MW1 TR-FRET conformational immunoassay can detect polyQ-dependent conformational effects in HTT protein in human HD, both centrally and peripherally.

### Serine phosphorylation in the N17 domain influences mutant huntingtin conformation

**Figure 9:**
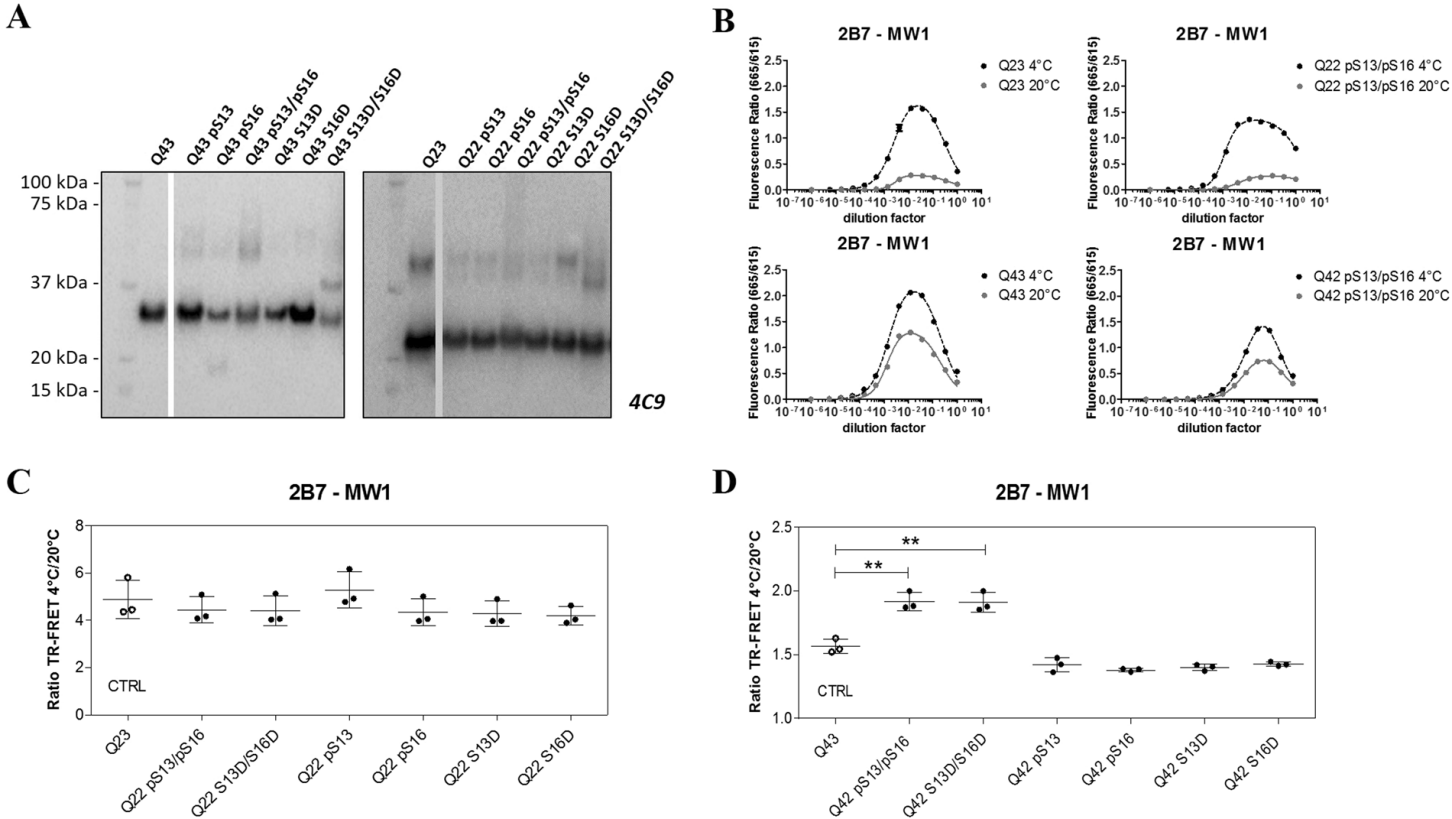
S13/S16 modifications in the N17 domain of HTT alter mHTT conformation as detected by the 2B7/MW1 conformational immunoassay. (**A**) Western blot of semisynthetic HTT exon 1 proteins (100 ng/lane; Q42/43 and Q23/22) used in this study (detection with mAb 4C9). WB images were cropped from the acquired original file. Full-length blots are reported in Supplementary Information: Figures S4 and S5. (**B**) Upper panels: 2B7/MW1 TR-FRET assay signal obtained on semisynthetic unmodified (Q23) or pS13/pS16 HTT exon 1 Q22 at the two temperatures. Lower panels: 2B7/MW1 TR-FRET assay signal obtained on semisynthetic unmodified (Q43) or pS13/pS16 HTT exon 1 Q42 at the two temperatures. (**C**) Summary of 2B7/MW1 conformational immunoassay data (ratio TR-FRET signals at 4 °C/20 °C) obtained on all wild type (Q23/22) semisynthetic proteins from three independent experiments. (**D**) Summary of 2B7/MW1 conformational immunoassay data (ratio TR-FRET signals at 4 °C/20 °C) obtained on all mutant (Q43/42) semisynthetic proteins from three independent experiments, each with three technical replicates (two-way ANOVA with Bonferroni’s post-test, degrees of significance are indicated).

**Figure 10:**
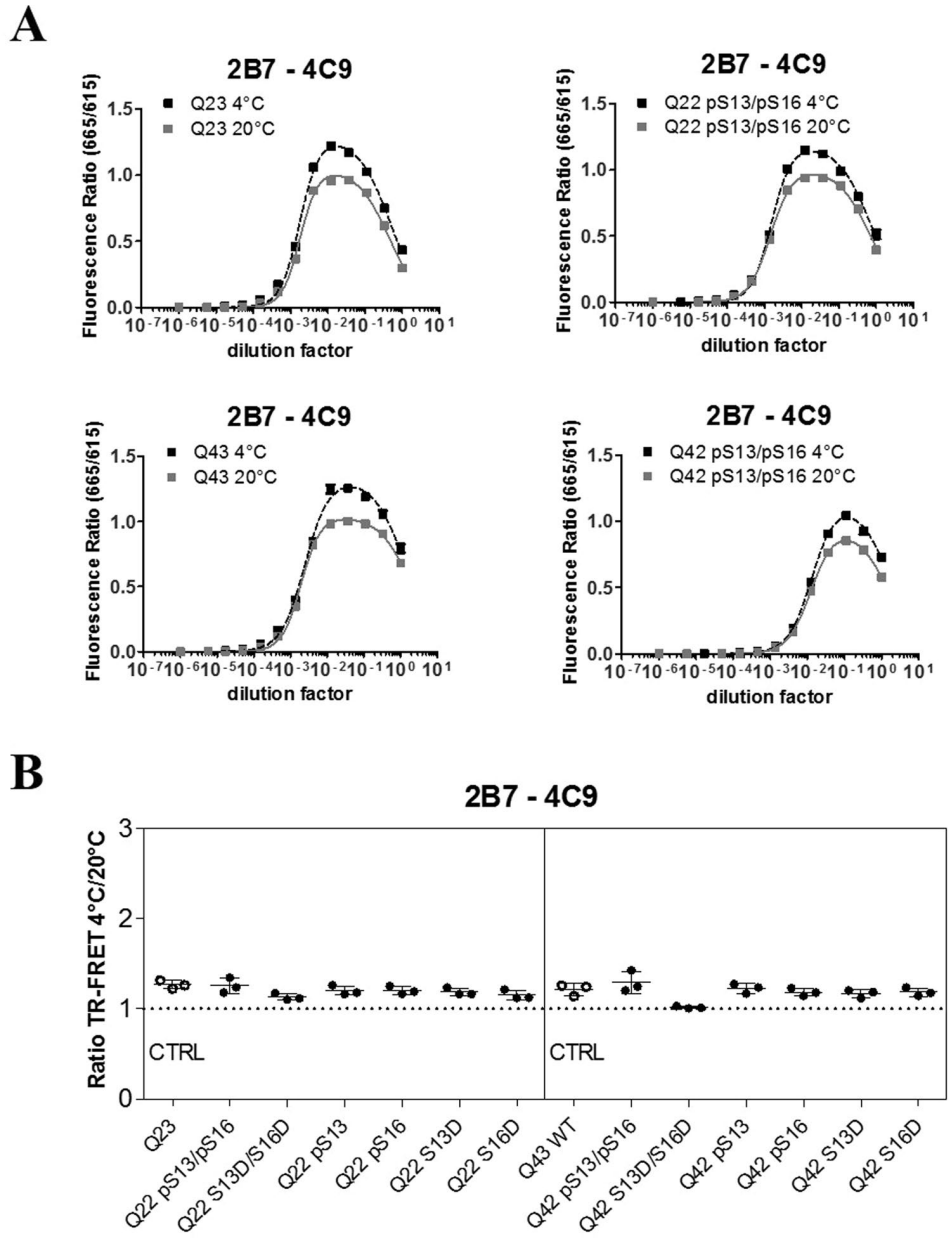
The 2B7/4C9 control immunoassay in semisynthetic HTT exon 1 proteins. (**A**) Upper panels: 2B7/4C9 TR-FRET assay signal obtained on semisynthetic unmodified(Q23) or pS13/pS16 HTT exon 1 Q22 at the two temperatures. Lower panels: 2B7/4C9 TR-FRET assay signal obtained on semisynthetic unmodified (Q43) or pS13/pS16 HTT exon 1 Q42 at the two temperatures. (**B**) Summary of 2B7/4C9 (control) TR-FRET immunoassay (ratio TR-FRET signals at 4 °C/20 °C), which does not detect the polyQ-dependent conformational change, obtained on all wild type (Q23/22) and all mutant (Q43/42) semisynthetic proteins from three independent experiments, each with three technical replicates (two-way ANOVA with Bonferroni’s post-test, degrees of significance are indicated).

Several studies suggest that phosphorylation (S13/S16 in particular) within the first 17 amino acids (N17 domain) of HTT affects critical biological properties of the protein, including subcellular localization, stability, aggregation, conformation and toxicity^10, 13–17^. Of these, the only study using HTT exon 1-like synthetic peptides with bona fide S phosphorylation is that of Mishra R *et al*.^17^, while most other studies were performed using HTT proteins bearing S13/16 mutations to either mimic (D or E) or abolish (A) phosphorylation, or using pharmacological modulation of cellular protein phosphorylation achieved through kinase inhibition. Previous studies by us^11^ and others^12^ indicated that compound phosphomimetic (S → D) or null (S → A) mutations at positions S13/S16 did not appear to alter HTT conformation, as measured by the conformational immunoassay, in either wild type or mutant HTT proteins expressed in cells. The recent availability of semisynthetic HTT exon 1 proteins bearing *bona fide* phosphorylation at specific residues within the N17 region^34^ prompted us to re-examine the conformational effects of S13/S16 HTT phosphorylation using purified semisynthetic proteins. We reasoned that controllable and defined conditions such as those attainable with isolated, purified proteins would better enable detection of smaller differences in conformation which might be difficult to detect in more complex samples (such as total lysates of cells). We elected to employ HTT exon 1 sequences bearing polyQ lengths of Q22 (representative of wild type HTT) and Q43, representative of a common mutant allele in HD^42, 43^. All semisynthetic proteins, either without or with phosphorylation and position S13, S16, S13 and S16 or bearing phosphomimetic (S → D) mutations at these positions for comparisons, were produced and quality controlled as described^34^. The HTT proteins were first resuspended with great care from lyophilized batches to reduce aggregation, and then examined by standard Western blotting (Fig. 9A). Although some evidence of protein aggregation was observed (variable amounts of immunoreactive material in the wells; Fig. S4), the vast majority of the HTT protein was resolvable on SDS-PAGE. Samples were then subjected to TR-FRET analysis using the 2B7/MW1 and 2B7/4C9 immunoassays at the two temperatures. As 2B7 recognizes an epitope within the N17 region of HTT, the 2B7/4C9 immunoassay served both as a conformational immunoassay control as well as a control TR-FRET assay for detecting interference with HTT N17 detection by 2B7 caused by S13/S16 phosphorylation. As shown in Figs 9B and 10A, both immunoassays efficiently detected the semisynthetic HTT proteins, with the expected temperature- and polyQ-dependent shift in TR-FRET signal produced by the 2B7/MW1 immunoassay (Fig. 9B) but not by the 2B7/4C9 assay (Fig. 10A). For simplicity, data pertinent to the analysis of HTT exon 1 peptides (Q22 and Q43) bearing no phosphorylation or the compound S13/S16 phosphorylation are shown. When the datasets obtained at the two temperatures with the 2B7/MW1 immunoassay were analyzed to investigate conformational effects, no significant variations arising from either single or compound phosphorylation at S13/S16 were observed for the wild type (Q22) HTT exon 1 protein, as was also the case for the single or compound phosphomimetic (S → D) mutations (Fig. 9C). Interestingly, however, a significant effect of the compound (S13 and S16) phosphorylation or the corresponding compound phosphomimetic mutations was observed when the 2B7/MW1 TR-FRET datasets relevant to the mutant (Q43) HTT protein were analyzed (Fig. 9D). The apparent effect of the compound phosphorylations or phosphomimetic mutations was to partly reduce the conformational constraint imposed by the mutant polyQ, an effect which was not produced by individual phosphorylation or phosphomimetic mutation at either S13 or S16 (Fig. 9D). Importantly, the control TR-FRET immunoassay (2B7/4C9) did not reveal any temperature- and polyQ-dependent change in signal at the two temperatures (Fig. 10B). Therefore, in the context examined N17 phosphorylation can alter mutant HTT conformation, at least partly ameliorating the conformational rigidity imposed by polyQ expansion. This may be the result of an alteration of N17 α-helicity, which is believed to be affected by polyQ expansion^44^.

## Discussion

Protein misfolding is thought to be directly associated with protein conformation, which is known to be influenced by various factors such as protein stability, inter- or intramolecular protein-protein interactions, and post-translational modifications. A number of rare (such as HD) as well as common (such as Alzheimer’s and Parkinson’s diseases) neurodegenerative pathologies can be defined as a “conformational diseases” due to the presence of misfolded and aggregated proteins arising from mutation and/or abnormal accumulation. In the case of HD, expansion of the polyQ tract is causative and is known to strongly affect the biophysical and biological properties of HTT. Aside from the well-known effect of mutant polyQ expansion on HTT oligomerization/aggregation^45–47^, recent findings using novel assays point to a significant conformational constraint imposed on HTT monomeric protein by the mutant polyQ expansion^10–12^. Owing to their intrinsic characteristics (they are homogeneous, robust, scalable and rapid; ref. 11) these novel TR-FRET based immunoassays represent a practical readout to identify and exploit genetic and/or pharmacological means that can ameliorate the conformational impact imposed on HTT protein by the mutant polyQ expansion. However, at least three critical aspects need to be addressed. First, the capacity of the conformational immunoassays to detect this conformational impact in preclinical HD models of increasing complexity, such as those that might be employed to obtain evidence of therapeutic efficacy *in vivo*. Second, the clinical relevance of the readout – specifically, the capacity of the conformational immunoassays to detect the polyQ-dependent conformational effect in humans in both peripheral as well as central compartments and to establish an association with disease onset/progression. Third, the demonstration that perturbations can be identified that have the potential to ameliorate the polyQ-dependent conformational impact in mutant HTT protein. Here, we aimed at starting to address these aspects demonstrating the capacity of the conformational TR-FRET based immunoassay to detect the polyQ-dependent conformational effect under conditions modeling heterozygosity (cocktails of isolated proteins, lysates of cells overexpressing both wild type and mutant HTT) or representing *bona fide* heterozygosity (fibroblasts expressing both alleles from the endogenous locus). These findings indicated that several options may be open to enable screening for genetic and/or pharmacological modulators of HTT Amino-terminal conformation, including the use of isolated proteins and different cellular models expressing HTT proteins. Next, the capacity of the conformational immunoassay to detect the polyQ-dependent conformational effect on HTT was demonstrated in Drosophila (TgHD) HD models. This indicated that it may be possible to test genes/compounds identified in screens for the capacity to alter the polyQ-dependent conformational impact on HTT protein in relevant animal models of HD. Collectively, the data address the first aspect raised above, and suggest that the conformational immunoassays can detect the polyQ-dependent conformational impact on HTT in preclinical HD models such as those that may be employed during preclinical efficacy studies. The second aspect raised above, pertinent to the relevance of the polyQ-dependent conformational effect as detected by the conformational immunoassay for human HD, was at least partially addressed by the detection of this effect in samples (lymphocytes, post-mortem brain tissue) from HD patients. Although the correlation between polyQ expansion-mediated conformational change imposed on HTT and pathology remains to be further investigated, the evidence in the literature strongly indicates that polyQ expansion-mediated conformational changes are involved in HD pathology. As the conformational TR-FRET immunoassay detects polyQ-dependent conformational changes in HTT, these findings are supportive of relevance for human HD, considering the well-known inverse correlation between polyQ length and age of onset^2, 46^. Finally, to address the third aspect raised above, we attempted the identification of perturbations that might influence the polyQ-dependent conformational constraint on huntingtin. We reasoned that focusing on modifications of the N17 domain, a region of the HTT protein well known for its propensity to be extensively post-translationally modified and to modulate various aspects of the biophysics and biology of huntingtin^10, 13–17, 48^ might represent a logical starting point. In particular, phosphomimesis at S13 and S16 has been shown to influence aggregation, toxicity, subcellular localization and conformation of mHTT^10, 13–16^. Indeed, we and others^11, 12^ previously attempted to investigate the influence of N17 S13 and S16 phosphomimetic mutations on HTT’s polyQ-dependent conformational constraint as detected by the conformational immunoassay in lysates from cells overexpressing HTT proteins. However, the relative complexity of the sample (total cell lysates) as well as the unknown degree to which phosphomimetic mutations phenocopy *bona fide* phosphorylation may have masked any effects, particularly when these were small. Instead, the effects of bona fide phosphorylations present on isolated, semisynthetic wild type or mHTT exon 1 proteins of defined composition were investigated. In this defined, controlled context a significant effect of compound S13 and S16 phosphorylation was observed on mutant (Q43) HTT conformation as detected by the 2B7/MW1 TR-FRET conformational immunoassay. Interestingly, this effect was comparable to that observed with compound S13/S16 phosphomimetic mutations, while none of the individual modifications (phosphorylation or phosphomimesis) on S13 or S16 produced an observable effect. Interestingly, it has been postulated that that S13 primes modification of S16 and that the majority of phosphorylation at these residues exists as a compound (pS13/pS16) event^16^. Based on previous work^11^, the observed S13/S16 modification-induced change in the conformational immunoassay readout might reasonably be due to a change (presumably a decrease, as these modifications apparently reduce the conformational signal gap between wild type and mHTT) in α-helical structure of the N-terminus of the protein. mHTT is apparently more conformationally rigid than wild type HTT^10–12^, possesses a higher α-helical content, and cooling increases its α-helicity more strongly than for wild type HTT^11, 49^. A decrease in the α-helicity content of the mutant protein may result in increased conformational flexibility, effectively reducing the gap between conformational immunoassay values observed for the wild type and mutant HTT proteins and explaining the observed increase in the conformational immunoassay readout. Consistently, an effect of S13/S16 modifications on N17 α-helicity has been previously reported^16^, while studies on longer HTT-like peptides have failed to reveal significant effects^17^. Although further work, for instance using sensitive structural methodologies such as electroparamagnetic resonance spectroscopy^50^, is required to characterize the structural effects of S13/S16 modifications on HTT exon 1 structure, we propose that S13/S16 modifications can alter HTT Amino-terminal conformation and that this perturbation is detectable, at least in isolated proteins, by the 2B7/MW1 TR-FRET conformational immunoassay. Although the observed S13/S16 modification-induced conformational effect in mHTT is relatively small and perhaps not easily detectable in biological samples (particularly given the transient and incompletely penetrant nature of protein phosphorylation), it serves as a proof-of-principle that means might be devised to alter mHTT amino-terminal conformation. Interestingly, previous work has reported increased pS13/pS16 HTT levels in the YAC (128) mouse transgenic model of HD following treatment with ganglioside GM1, resulting in amelioration of the HD phenotype^18^. This is consistent with the effects of the phosphomimetic mutations S13D/S16D in another transgenic HD model^15^. It would be interesting to evaluate the effects of GM1 on HTT conformation in (YAC) 128 HD mice, as measured by the conformational TR-FRET immunoassay. This would address the possibility that the beneficial effects of GM1 on the HD phenotype in these mice may be at least in part due to an amelioration of mutant HTT conformational rigidity. Collectively, the data support the potential utility of the TR-FRET based conformational immunoassay as a tool to identify and develop genetic and/or pharmacological means to modify mHTT’s polyQ-induced conformational constraint, to further examine its role in HD pathogenesis and, potentially, to develop novel therapeutic approaches.

## Methods

### Plasmid constructs and proteins

cDNAs encoding amino-terminal fragments (exon 1, N548) or full length human HTT bearing different polyQ lengths were described previously^11^. Semisynthetic HTT exon 1 proteins (Q23, Q43) with/without relevant S13/S16 modifications were produced and quality-controlled essentially as described^34^. Recombinant human proteins containing the amino-terminal sequence of HTT with 573 amino acids (N573)^23^ and full length HTT with polyQ repeats of either wild type or mutant lengths were kindly provided by CHDI Foundation.

### Cell lines

HEK293T cells were cultured in DMEM, 10% FBS, 1% Penicillin and Streptomycin (all from Gibco by Life Technologies) as per supplier’s instructions. Cells were routinely transfected with plasmid constructs using Lipofectamine 2000 (Life Technologies) as per manufacturer’s instructions. Twenty-four hours after transfection cells were harvested and lysed in lysis buffer (PBS, 0.4% Triton X-100) supplemented with 1X protease inhibitor cocktail (Roche).The fibroblast cell lines are listed in the table reported in Fig. 5A. They were obtained from NIGMS Human Genetic Cell Repository at the Coriell Institute for Medical Research. Cells were cultured in MEM, 15% FBS, 1% Penicillin and Streptomycin (all from Gibco by Life Technologies) in accordance with the supplier’s instructions, harvested, and lysed in lysis buffer (as reported previously for HEK293T cells).

### Drosophila tissues

Drosophila tissues were processed as previously described^9^. Single wandering third instar larvae were ground for 1 min with an electric pestle in 30 ml PBS and combined with 30 ml 2x loading buffer.

### Human samples

A program to collect biological specimen at IRCCS Casa Sollievo della Sofferenza and at Mendel Institute of Human Genetics for research purposes, including blood samples, was approved by the Ethical Committee from Casa Sollievo della Sofferenza Foundation, section of Istituto Tumori Giovanni Paolo II in Bari (Italy). Informed consents was obtained from patients and healthy control subjects. Recruitment of control and HD subjects was performed as described in ref. 51. A total of 11 HD subjects (stage I-IV), and 3 gender- and age-matched healthy controls were recruited. Subjects’ demographic, clinical and genetic characteristics are reported in Fig. 7A. All HD subjects revealed a CAG repeat expansion mutation, and all of them as well as controls were required to sign an informed consent before recruitment in the study. All human experiments were performed in accordance with the Declaration of Helsinki. Peripheral blood mononuclear cells (PBMCs) were obtained by standard methodologies, as described^51^ and cell pellets were immediately snap frozen. Total homogenate protein concentration was determined with BCA assay (Thermo Scientific). Post-mortem brain tissue (cortex) from ten patients at different pathological grades of HD^52^ and three healthy controls were examined in this study. Samples were obtained by the New York Brain Bank at Columbia University, New York, USA. Clinical and neuropathological data were summarized in Fig. 8A. They were transferred in 1x lysis buffer (1x PBS, 0.4% Triton X, 1x protein inhibitor cocktail, Roche), in ceramic beads containing vials (Lysing matrix D, MP Biomedicals, cat. n. 116913050) and homogenized with a FastPrep96 homogenizer (3 × 30 seconds cycles at 1600 rpm). Samples were kept at −80 °C overnight and clarified before total protein quantification.

### Antibodies

The MW1 antibody was developed by Dr. Paul Patterson^30^ and obtained from the Developmental Studies Hybridoma Bank developed under the auspices of the NICHD and maintained by The University of Iowa, Department of Biological Sciences, Iowa City, IA 52242. 2B7 and 4C9 antibody generation and characterization were described elsewhere^33, 53^. Purified 2B7, 4C9 and MW1 antibodies were obtained through the CHDI Foundation (New York, NY). Custom terbium cryptate and D2-fluorophore antibody labeling was performed by CisBio (Bagnols, France) as described previously^11^. Antibody MAB2166 was from Millipore (cat. n. MAB2166).

### Western blot assay

Total protein levels of lysates obtained from HEK293T cells and Drosophila HD model, the latter produced as in ref. 9, were quantified using the Pierce BCA Protein Assay kit (Thermo Scientific) according to manufacturer’s protocol. 20 µg of total lysates were denatured at 95 °C in 4x loading buffer (125 mM TrisHCl pH 6.8, 6% SDS, 4 M urea, 4 mM EDTA, 30% glycerol, 4% β-mercaptoethanol and Bromophenol blue) and loaded on NuPAGE 4–12% Bis-Tris Gel or WedgeWell 6% Tris-Glycine Gel (Life Technologies). Proteins were transferred on PVDF membranes using the transfer apparatus from Life Technologies or Trans-Blot Turbo Transfer System from Biorad following manufacturer’s protocol. Membranes were stained for 30 minutes in TBS, 0.1% Tween, 0.4% PFA, before 1 hour blocking in TBS, 0.1% Tween, 5% not fat milk. Incubations with primary antibodies were performed overnight at 4 °C. Incubations with secondary anti-mouse- or rabbit horseradish peroxidase conjugated antibodies were carried out for 1 hour at room temperature. Protein bands were revealed using chemiluminescence substrate (ECL from Life Technologies) and images were acquired using a Chemidoc imager (Biorad). Similarly, synthetic huntingtin exon 1 proteins were loaded on NuPAGE 4–12% Bis-Tris Gel (Life Technologies) for Western blotting analysis.

### TR-FRET assays

TR-FRET assays were performed as described previously^11^. In brief, samples were transferred to a low volume 384 well plate (Greiner) in serial dilutions starting from a defined concentration (1–4 µg/µl for total lysates and homogenates and 2 ng/µl for recombinant proteins) and the equivalent of one fifth of sample volume of antibody cocktail was then added. Soluble HTT was measured with 2B7-Tb/MW1-D2 and 2B7-Tb/4C9-D2 using 0.17 ng/µl of 2B7-Tb and 1.7 ng/µl of D2 labelled antibody. TR-FRET measurements were routinely performed following 1 hour incubation at 20 °C or overnight at 4 °C using an EnVision Reader (Perkin Elmer). Data analysis and evaluation of the temperature- and polyQ-dependent variation in TR-FRET signal were performed as previously described^11^. The points in the curves correspond to the averages of the background-subtracted fluorescence ratio (665/620) relative to the samples replicates and the bars represent the standard deviation among these replicates. The bar graphs represent the temperature- and polyQ-dependent variation and the standard deviations among values obtained in different experiments.

### Statistics

Normality test, Student’s t-tests and One-way or Two-way ANOVA were performed to calculate the significance amongst repeated measurements (*p < 0.05; **p < 0.01; ***p < 0.005; ****p < 0.001) using GraphPad Prism software. For each experiment at least three independent replicas were analyzed.

## Electronic supplementary material

Supplementary information
