# Supplementary material for: Polyglutamine expansion affects huntingtin conformation in multiple Huntington’s disease models
Source: Sci Rep. 2017 Jul 11;7:5070. doi: 10.1038/s41598-017-05336-7 (PMC5505970; doi:10.1038/s41598-017-05336-7)
Supplement: Supplementary file 1 — Supplementary information [file 41598_2017_5336_MOESM1_ESM.pdf]

Polyglutamine expansion affects huntingtin conformation in multiple Huntington's disease models

Manuel Daldin[1+], Valentina Fodale[1,2+], Cristina Cariulo[1], Lucia Azzollini[1,2], Margherita Verani[1,2], Paola Martufi[1], Maria Carolina Spiezia[1], Sean M. Deguire[3], Marta Cherubini[4], Douglas Macdonald[5], Andreas Weiss[1,#], Alberto Bresciani[2], Jean-Paul Gerard Vonsattel[6], Lara Petricca[1], J. Lawrence Marsh[7], Silvia Gines[4], Iolanda Santimone[8], Massimo Marano[8], Hilal A. Lashuel[3], Ferdinando Squitieri[8] and Andrea Caricasole[1,2*]

Supplementary information

Supplementary figure S1

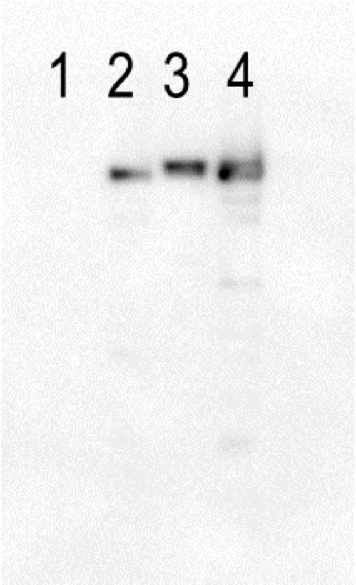

Full-length blot image used for figure 4 A. Representative Western blotting experiments of HEK293T cell lysates obtained by transient transfection with plasmids encoding individual full length HTT proteins, wild type or mutant, or a cocktail of plasmids encoding both, probed with antibody 4C9 (detecting HTT).

Lane legend:

1. Empty vector (pCDNA 3.1)
2. HTT FL Q23
3. HTT FL Q73
4. HTT FL Q23+Q73

Supplementary figure S2

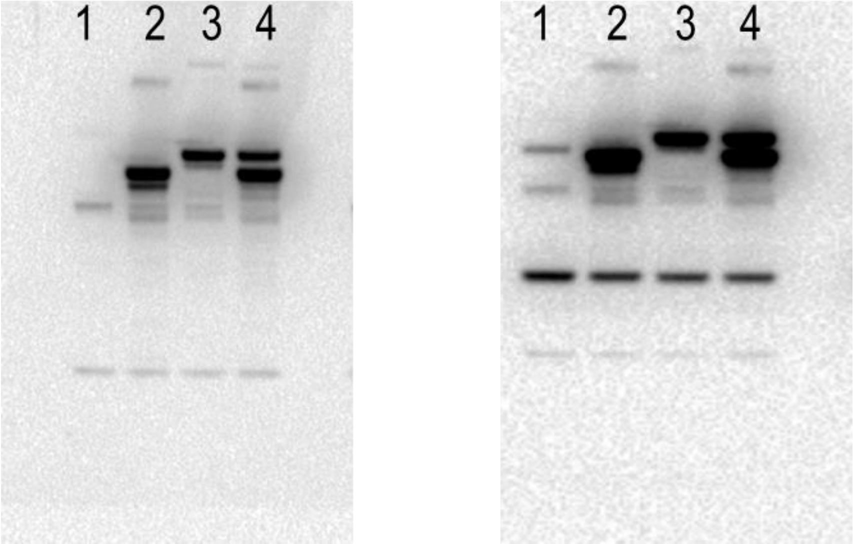

N548 blot image used for figure 4 B. Representative Western blotting experiments of HEK293T cell lysates obtained by transient transfection with plasmids encoding individual N548 HTT proteins, wild type or mutant, or a cocktail of plasmids encoding both, probed with antibody 4C9 (detecting HTT – figure on the left) and GAPDH (as loading control - figure on the right).

Lane legend:

1. Empty vector (pCDNA 3.1)
2. HTT N548 Q16
3. HTT N548 Q55
4. HTT N548 Q16+Q55

Supplementary figure S3

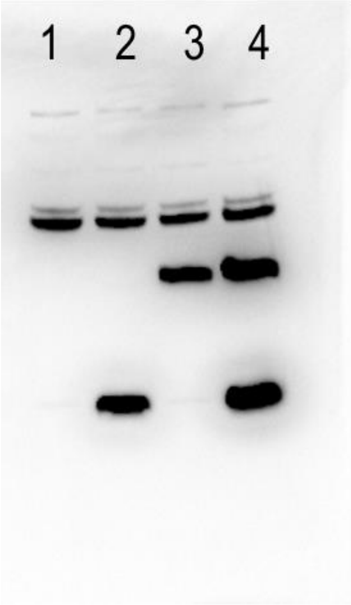

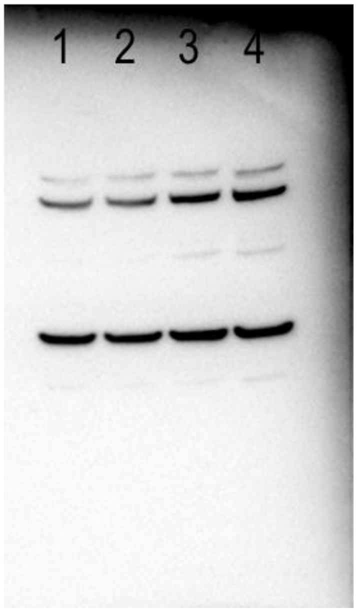

Full-length blot image used for figure 4 C. Representative Western blotting experiments of HEK293T cell lysates obtained by transient transfection with plasmids encoding individual exon 1 HTT proteins, wild type or mutant, or a cocktail of plasmids encoding both, probed with antibody 4C9 (detecting HTT – figure on the left) and GAPDH (as loading control - figure on the right).

Lane legend:

1. Empty vector (pCDNA 3.1)
2. HTT Ex1-Q16
3. HTT Ex1-Q72
4. HTT Ex1-Q16+Q72.

Supplementary figure S4

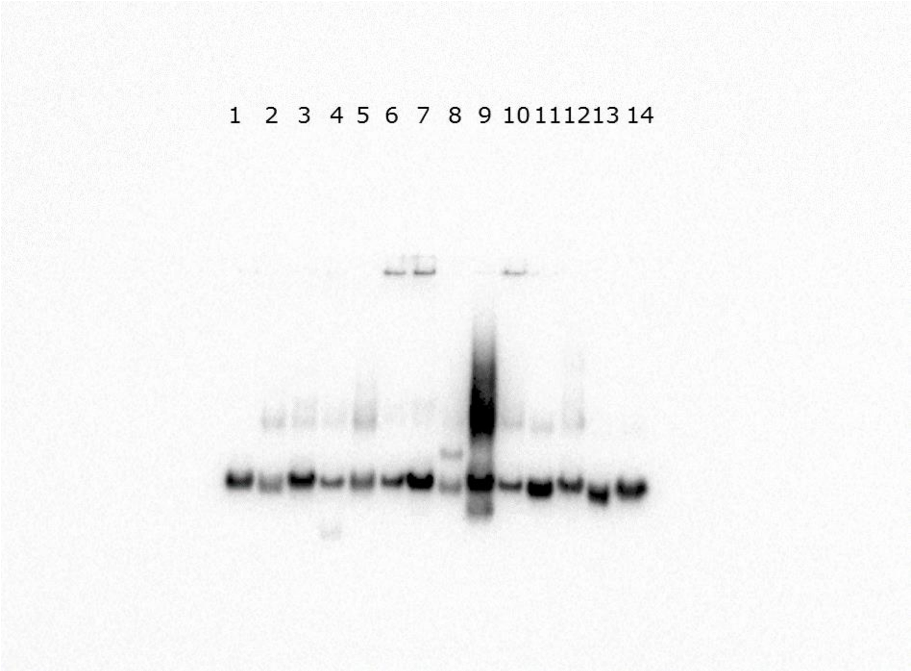

Full-length blot image used for the left part of figure 9 A. Western blot of semisynthetic HTT exon 1 Q43 proteins (100 ng/lane; Q43 and Q22) used in this study (detection with mAb 4C9).

Lane legend:

1. Q43
2. Q43 pT3
3. Q43 pS13
4. Q43 pS16
5. Q43 pS13/pS16
6. Q43 S13D
7. Q43 S16D
8. Q43 S13D/S16D
9. Q43 Ack6
10. Q43 Ack9
11. Q43 T3D
12. Q43 pT3/Ack6
13. Q43 Ack6
14. Q43 Ack9

Images reported in the left part of figure 9 A including lanes 1, 3, 4, 5, 6, 7, and 8; samples in lanes 2, 9, 10, 11, 12, 13, and 14 are irrelevant for the manuscript and not cited anywhere.

Supplementary figure S5

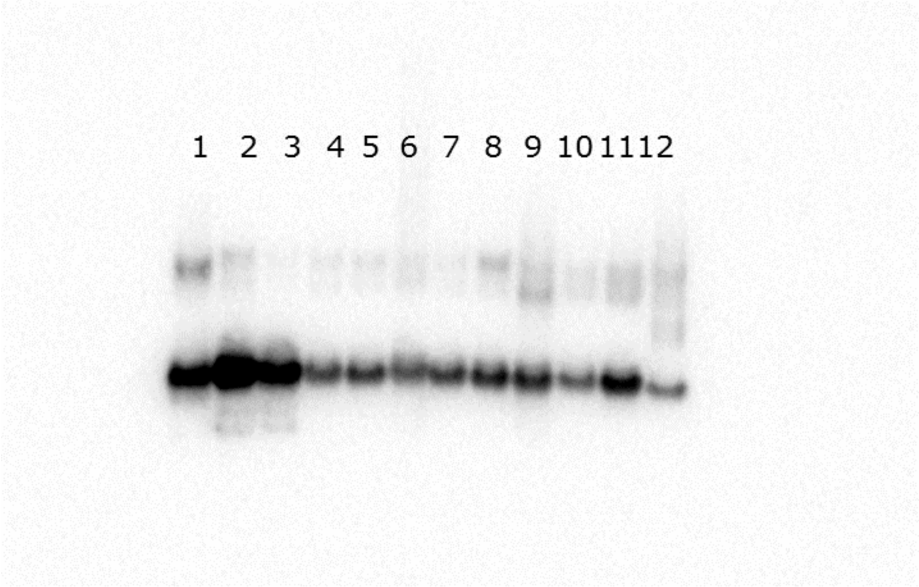

Full-length blot image used for the right part of figure 9 A. Western blot of semisynthetic HTT exon 1 Q22 proteins (100 ng/lane; Q43 and Q22) used in this study (detection with mAb 4C9).

Lane legend:

1. Q22
2. Q23
3. Q23 pT3
4. Q22 pS13
5. Q22 pS16
6. Q22 pS13/pS16
7. Q22 S13D
8. Q22 S16D
9. Q22 S13D/S16D
10. Q23 Ack6
11. Q23 Ack9
12. Q23 Ack15

Images reported in the right part of figure 9 A including lanes 1, 4, 5, 6, 7, 8 and 9; samples in lanes 2, 3, 10, 11 and 12 are irrelevant for the manuscript and not cited anywhere.

Supplementary figure S6

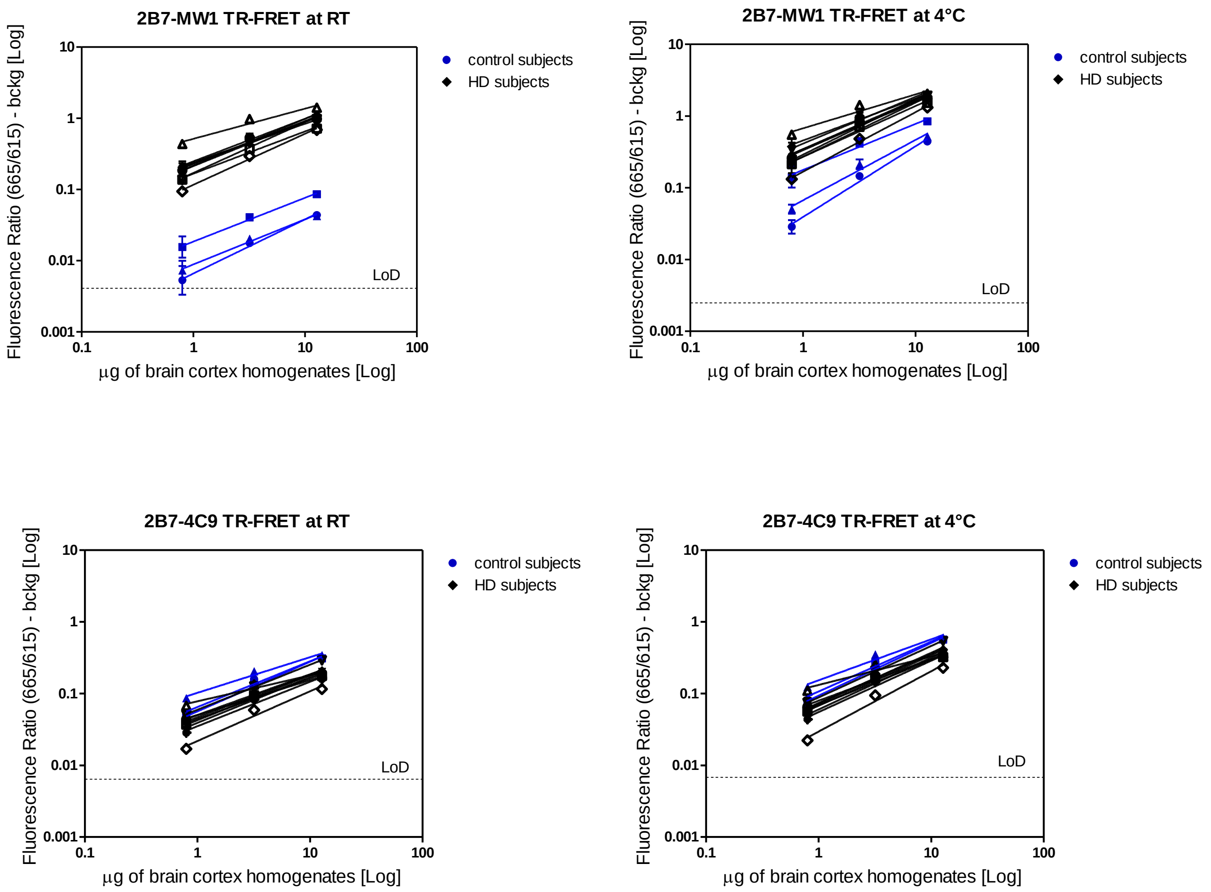

Fluorescence signals obtained from the 2B7/MW1 (upper two graphs) and the 2B7/4C9 (lower two graphs) TR-FRET analysis of control and HD post-mortem human brains (cortex), performed at room temperature (two graphs on the right) and 4°C (two graphs on the left).

Three samples from control subjects and ten samples from HD subjects were analyzed in three dilution points starting from 12 μg of homogenates total protein. In order to perform the conformational immunoassays, the obtained signal point series were fitted with straight lines and the Y0 intercept value, of each line at each of the two tested temperature, was used to perform the ratio reported in figure 8 B, C and D, which is the readout value of the conformational immunoassay.
